# Spectral Response of Metallic Optical Antennas Driven by Temperature

**DOI:** 10.1007/s11468-016-0297-z

**Published:** 2016-06-15

**Authors:** Alexander Cuadrado, José Manuel López-Alonso, Francisco Javier González, Javier Alda

**Affiliations:** 1 Laser Processing Group.Insitute of Optics. Consejo Superior de Investigaciones Científicas, C/ Serrano 121, 28006 Madrid, Spain; 2 Applied Optics Complutense Group, University Complutense of Madrid, C/ Arcos de Jalón, 118, 28037 Madrid, Spain; 3 Coordinación para la Innovación y Aplicación de la Ciencia y la Tecnología, Universidad Autónoma de San Luis Potosí, Sierra Leona, 550, Lomas 2a Seccion, CIACYT Building, 78210 San Luis Potosí, SLP México

**Keywords:** Optical antennas, Spectrometer

## Abstract

When optical antennas are used as light detectors, temperature changes their spectral response. Using this relation, we determine the spectrum of a light beam from an optical antenna’s signal. A numerical evaluation of the temperature-spectral response has been completed with a model for the noise of the device. Using both the response and the noise model, we have established the capabilities of the device by quantifying the error in the spectrum determination both for broadband spectrum and monochromatic radiation.

## Introduction

Optical antennas can act as light detectors. They present some advantages linked to the nature of their physical mechanism of operation. Optical antennas are selective to polarization, spectral content, and directional angular patterns. Moreover, they have a receiving area smaller than *λ* ^2^ [1–4]. Plasmonic optical antennas have transferred the technology that is of common use in the RF and microwave frequency domains with promising results. One of the devices that have demonstrated to be complicated to transfer to the optical domain is the tunable antenna. This is due to the fact that at higher frequencies, the losses are so considerable that a conventional capacitive or inductive element would not affect the response of an antenna at optical frequencies. By using an increase in temperature to tune the frequency response of an optical antenna, it is possible to incorporate to the optical domain systems that are of common use at lower frequencies but have not been realized at optical frequencies such as optical phased arrays [5]. To generate an electric signal readable by an external circuit, optical antennas need transduction mechanisms [6]. For example, the rectification of currents built up in the resonant element is typically done by metal-insulator-metal junctions (MIM) [7]. The device’s temperature also changes because of Joule dissipation, producing a bolometric effect that is exploited in antenna-coupled bolometers [8, 9]. In bimetallic antennas, the change in temperature induces a Seebeck voltage [10, 11].

External biasing is of importance in MIM devices and bolometers and it also can be considered as a driving parameter in optical antennas [7, 12]. Besides, photon-electron interactions and thermal mechanisms affect the generation of hot carriers within the resonant element. These contributions modify the signal and also the integrity of optical antennas [13]. A catastrophic event is typically triggered by a temperature increase at the location of the antenna and connecting lead lines. Changes in morphology due to dewetting can be produced by high power pulses or by long periods of high temperature, and those limits should be considered when exposing optical antennas to irradiance or Joule heating [14, 15]. For moderate irradiance values, Joule dissipation from optical radiation is secondary when considering the robustness of optical antennas [16]. If temperature is below damage threshold, it still changes the optical properties of materials. Thus, it can be taken as a parameter to control the response of the device. In most cases, the same external bias circuit that generates the output signal can change the temperature and drive the device electronically [16]. By selectively changing the temperature within a dipole antenna array, it is possible to induce a change in the relative phase between resonant structures and steer optical antennas [5]. Previous contributions have studied the role of the bias source and the influence of the geometry of the bias structures [17] on the response of an antenna. It has been proved that when the bias voltage exceed a limit value, the device is compromised and fails [18]; and before breakdown, it may induce non-linear mechanisms [19]. Another way to heat the device is with plasmonic structures placed around the dipole antenna [20]. The rise in temperature would be caused by another light source delivering enough power to the device. Therefore, the response of an optical antenna is temperature-dependent and temperature can be controlled by using, for example, a bias voltage.

In this paper, we propose the use of temperature as a control parameter to tune optical antennas and change its spectral response. Thermochromic metamaterials have already demonstrated how temperature can control spectral and phase displacement in plasmonic devices [21]. The design presented here uses a single optical antenna as an IR spectrometer with a very high spatial resolution and polarization sensitivity. The spectral region of interest is the long wave infrared window, 8–12 μm (LWIR). In Section Spectral Response vs. Temperature, we analyze the response driven by temperature for bolometric devices. To complete the study, we have evaluated the noise contributions applicable to these detectors and how noise affects the determination of the spectrum. Section Spectrum Evaluation illustrates how the device works when different spectral distributions impinge on the optical antenna. A detailed analysis of the spectrum retrieval process accounts for noise. We have also analyzed the case of monochromatic spectra. Finally, in Section Conclusions, we summarize the main findings of this paper.

## Spectral Response vs. Temperature

**Fig. 1:**
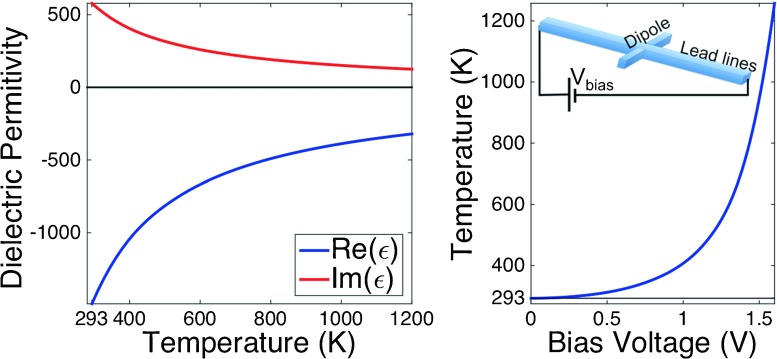
**a** Variation of the real and imaginary part of the dielectric constant of nickel with temperature at *λ* = 10.6μm. **b** Temperature of the dipole antenna as a function of the bias voltage, *V*_*b*_. The *inset* shows a sketch of how *V*_*b*_ can be applied to the dipole antenna

We found that the variation of dielectric permittivity of metals with temperature is responsible for the change in response of an optical antenna with temperature. Metals behave as non-perfect conductors at optical frequencies and the optical properties in this regime are also temperature dependent. As an example, in Fig. 1a, we show how both the real and imaginary parts of the dielectric constant of nickel change with temperature. Consequently, these changes modify the penetration depth within the resonant structures and vary the resonance of the dipole.

The electric field incident on an optical antenna may have a spectral composition **E**(*ω*). This spectral distribution generates a current given by Ohm’s law: **J**(*ω*) = *σ*(*ω*)**E**(*ω*). In a bolometric device, this current dissipates as Joule heat and produces a change in temperature of the device that can be sensed externally. If the transducer is a MIM tunnel junction, the current through the junction will produce the signal. In both cases, electric conductivity, *σ*, is the key factor to understand the response of the device. From the Drude model, this parameter is related to the electric permittivity, *𝜖*(*ω*) = *𝜖* ^′^(*ω*) + *i𝜖* ^″^(*ω*), by the following equation:

1 $$ \sigma(\omega)=\omega \epsilon_{0} \left( \epsilon^{\prime\prime}(\omega) - i \left( \epsilon^{\prime}(\omega) -1 \right) \right) , $$

where *𝜖*_0_ is the dielectric constant of vacuum. As far as the dielectric constant is dependent on temperature, the conductivity varies with temperature. Then, generated currents become temperature-dependent.

For MIM antenna-coupled devices, besides conductivity, other temperature-dependent parameters are also involved in the transduction [22], specially when temperature spans on several hundreds degrees. Temperature changes the response of the MIM diode because of changes in the Fermi level produced by the increase of mobility [23, 24]. MIM junctions rectify the currents flowing through them [7]. MIM junctions are typically placed at the feed point of the antenna as a thin insulation layer. In this case, the rectified current density arriving to the junction could be given as the component across the junction, *J*_*z*_:

2 $$ J_{z}= \frac{1}{S} {\int}_{S} \sigma E_{z} ds , $$

where the average is made across the transverse section of the junction, *S*, and we only consider the *z* component of the electric field at the junction, *E*_*z*_. The signal delivered by the device is obtained from the current excited by optical radiation, *J*_*z*_, considering the characteristics of the junction (biasing, temperature, geometry, material parameters, and charge carrier’s dynamics) [7].

Previous work has demonstrated that one of the simplest ways to detect incoming radiation by using an antenna is by incorporating a bolometer into it [8, 9, 25]. This antenna-coupled transductor works by increasing its temperature through Joule heating generated by the induced current in the antenna; this will give a signal that is proportional to the incoming radiation. By using a bolometer coupled to an antenna, it is possible to make faster detectors since the antenna would be used to couple the radiation which is usually done by the surface of the bolometer. The total power absorbed by the resonant element changes the temperature of the device. To simplify the device even further, it is possible to distribute the bolometric effect along the whole antenna structure. The response of optical antennas working as distributed bolometers is sensed as a voltage variation across the detector [9]. In this case, the dissipated power is given as

3 $$ Q= {\int}_{v} \mathbf{J}^{\ast} \mathbf{E} dv= {\int}_{v} \sigma(T,\omega) | E(\omega) |^{2} dv , $$

where the integration is carried out within the whole volume of the antenna, *v*.

**Fig. 2:**
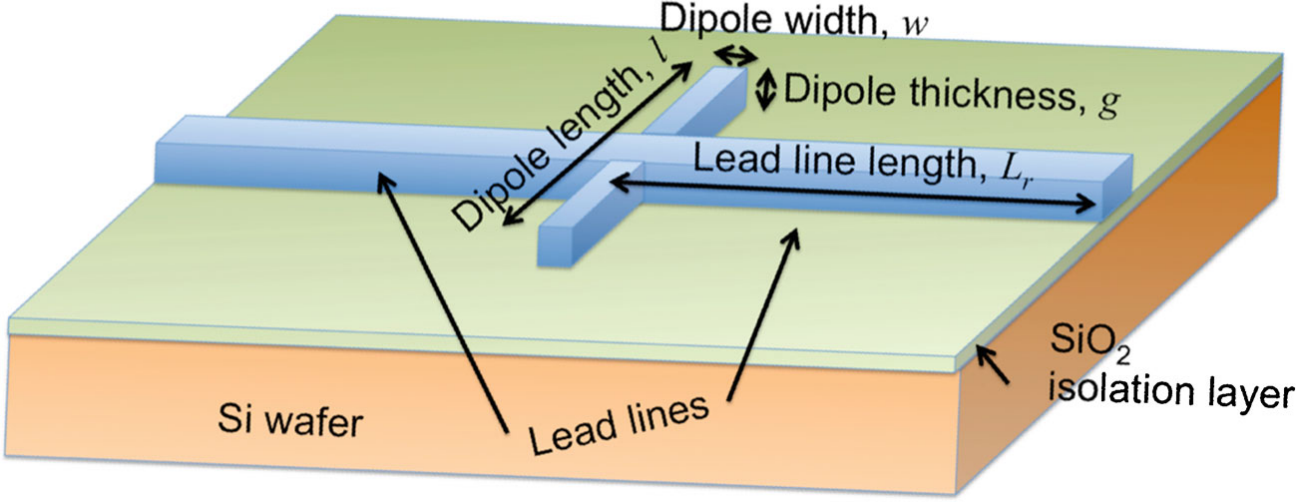
Geometrical arrangement of the dipole

**Fig. 3:**
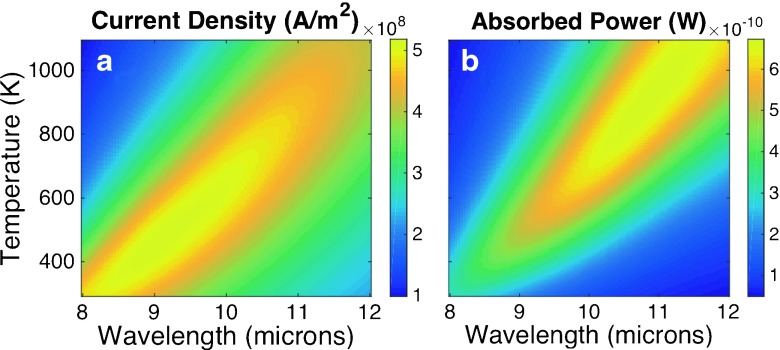
Current density flowing through the center of the dipole antenna (**a**) and total dissipated power at the antenna volume (**b**) as a function of wavelength and temperature. The current is calculated to model an antenna-coupled MIM diode, and the total dissipated power is valid for a distributed bolometer. The current density and total dissipated power are obtained using Eqs. (2) and (3). The antenna is a dipole oriented along the direction of an incident electric field with an amplitude of 220 V/m. A sketch of the dipole is given in Fig. 2

We have illustrated this dependence with temperature and wavelength analyzing a nickel dipole antenna perpendicularly oriented with respect to a load line (see Fig. 2). The dimensions of the dipole are 2.5 × 0.2 × 0.05 μ*m* ^3^ (length × width × thickness). This geometry is optimized to properly cover a spectral range between 8 and 12 μm. The lead lines have the same thickness of 50 nm and a width of 300 nm. The dipole is placed on a SiO _2_ layer of 1.2 μm in thickness. The substrate is modeled as a Si wafer. This element works as a MIM rectifier if both arms of the dipole are electrically isolated by a thin oxide layer. If the element is fabricated in a single deposition step, the antenna can be considered as a distributed bolometer [9]. Figure 3a shows the variation of the density current through the feed point of this dipole antenna configuration, applicable to MIM antenna-coupled detectors, *J*_*z*_ (Eq. (2)). Figure 3b represents the total dissipated power, *Q* (Eq. (3)), valid for distributed bolometers. These maps are obtained with COMSOL Multiphysics and express the dependence of these magnitudes with temperature and wavelength.

Same metal MIM junctions and bolometers require biasing to operate. In our case, this bias voltage, *V*_*b*_, is also the heating source. The value of the temperature reached by the nanoantenna as a function of the bias voltage is shown in Fig. 1b. Due to the small thermal inertia of the device associated with its tiny size, these changes can be made in very short time, allowing a high modulation frequency in the order of kHz [5]. From previous calculations, temperature drops two orders of magnitude when moving less than 1 μm away from an optical antenna on a SiO _2_ insulation layer (see Fig. 2 in reference [5]). This means that temperature changes are confined to the close vicinity of the resonant elements.

Response of MIM junctions is dependent on temperature, geometry of the junction, and material parameters in a more complicated way than bolometers [23, 24]. Therefore, for simplicity, we focus our attention on distributed bolometer optical antennas. It is known that bolometers produce a signal proportional to the optical irradiance which is dissipated as heat by the resonant structure [25]. The change in resistivity vs. temperature (bolometric effect) is sensed through a biasing circuit. If we know the temperature distribution, it is possible to obtain the signal produced by the optical radiation impinging on the antenna. A simple phenomenological model can be given to obtain the temperature profile along the lead line [9]:

4 $$ T(x)- T^{*} = \frac{Q_{b}}{2\kappa} \left\lbrace \begin{array}{ll} -x^{2} - \frac{w^{2}}{4}+ wL_{r} & \text{ if } |x|\leq \frac{w}{2} \\ -w|x|+wL_{r} & \text{ if } \frac{w}{2} < |x| \leq L_{r} \end{array}\right., $$

where *w* is the width of the dipole, *L*_*r*_ is the length along the load line to reach a location where the temperature is the operational temperature of the device, *T* ^∗^, *κ* is the thermal conductivity of the metal, and *Q*_*b*_ is the averaged absorbed power per volume unit, i.e., a power density. We obtain *Q*_*b*_ from the total dissipated power of the antenna as *Q*_*b*_ = *Q*/*v*, where *Q* is given by Eq. (3) and *v* is the volume of the antenna. The temperature of operation of the device, *T* ^∗^, is set by the biasing voltage (or current). This bias voltage (or current) must remain stable during measurement at the given temperature. Moreover, at each operational temperature, *T* ^∗^ should be stable with respect to the variation in temperature caused by optical irradiance.

The bolometric signal can be given as follows:

5 $$ {\Delta} V = I_{b} {\Delta} R = \frac{V_{b}}{R} {\Delta} R, $$

where Δ*R* describes the change in resistance due to the temperature profile, *T*(*x*), produced by the optical power absorbed by the dipole antenna, *R* is the resistance at the operational temperature *T* ^∗^, and *I*_*b*_ and *V*_*b*_ are the bias current and bias voltage, respectively. The equations for Δ*R* and *R* are as follows:

6 $$\begin{array}{@{}rcl@{}} {\Delta} R & = & \frac{\rho_{0} \alpha}{S} {\int}_{-L_{r}}^{L_{r}} T(x)dx \end{array} $$

7 $$\begin{array}{@{}rcl@{}} R & = &\frac{\rho_{0} L_{r}}{S} [1+ \alpha(T^{*}-T_{0})] \end{array} $$

where *T*_0_ is the temperature at which the resistivity of the material is *ρ*_0_, *α* is the coefficient of resistance with temperature (TCR), *S* is the transverse area of the load line, and *L*_*r*_ is the length from the dipole to the location where temperature reaches *T* ^∗^. The integration means that each portion of the lead line contributes in series to the change in resistance. In this equation, we consider that the material of the load lines and the dipole is the same, which is the case for distributed bolometers fabricated with a single material deposition.

Using the previous assumptions and the analytic solution for the temperature profile (see Eq. (4)), it is possible to obtain an expression for the bolometric signal, *V*_*s*_:

8 $$ V_{s}={\Delta} V = V_{b} Q_{b} \frac{\alpha}{\kappa} \frac{w}{4L_{r}} \left( {L_{r}^{2}} - \frac{w^{2}}{12} \right) = V_{b} Q_{b} \gamma . $$

**Fig. 4:**
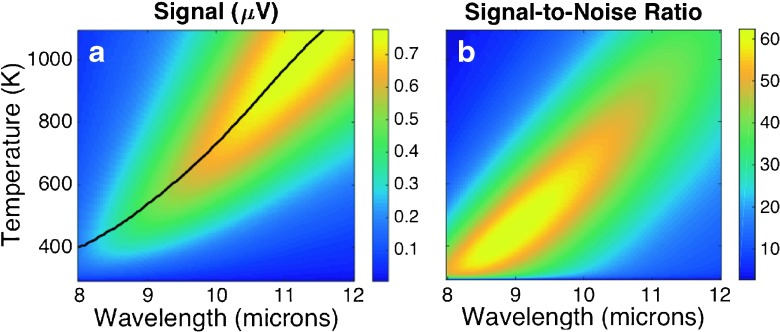
**a** Signal from the bolometric response of a dipole antenna using Eq. (8) The *black line* in this plot represents the location of the maximum for each *λ*. **b** Signal-to-noise ratio (SNR) for the dipole antenna. The incident radiation is a monochromatic plane wave linearly polarized along the direction of the dipole and with an electric field amplitude of 220 V/m

This signal, *V*_*s*_, is sensed as a variation with respect to the bias voltage, *V*_*b*_. In Eq. (8), we distinguish three main contributions: the bias voltage, *V*_*b*_, that sets the temperature of operation, *T* ^∗^; the absorbed optical irradiance given by *Q*_*b*_; and finally, a parameter, *γ*, that summarizes the geometrical and material characteristics. If we consider *w*≪*L*_*r*_ then *γ* = (*α*/*κ*)(*L*_*r*_ *w*/4). The bolometric signal, *V*_*s*_, obtained from the device is plotted in Fig. 4a, as a function of wavelength and temperature.

### Noise Evaluation

To operate the device, the antenna is heated to a certain operational temperature, *T* ^∗^, that is larger than the ambient temperature, *T*_0_. In these conditions, the identification of noise sources is very important to evaluate the operational mode and the capabilities of this device as a spectrometer.

When the bolometric effect is the transduction mechanism, the noise of the element can be modeled by considering thermal noise, temperature noise, and Johnson noise. These contributions depend on the geometry of the detector, the properties of the material (electric and thermal conductivities), and its temperature [26]. Johnson noise acts as a voltage source:

9 $$ V_{\text{{\small Johnson}}}=\sqrt{4 k_{B} T^{*}R{\Delta} f} , $$

where *k*_*B*_ is the Boltzman constant, Δ*f* is the bandwidth of the detection system, and *R* is the resistance at *T* ^∗^ given by Eq. (7). Thermal noise is produced by the heat exchange between the device at a temperature *T* ^∗^, and its surroundings at *T*_0_. This term is given as a noise equivalent power, NEP:

10 $$ \text{NEP}_{\text{{\small therm}}} = \sqrt{\frac{8k_{B} A_{d} \sigma_{\text{SB}} {\Delta} f ({T^{*}}^{5}+{T_{0}^{5}})}{\epsilon}} , $$

where *σ*_SB_ is the Stefan-Boltzman constant, *A*_*d*_ is the detector area, and *𝜖* is the emissivity of the device. The last contribution that we consider is due to the temperature fluctuations in the device. This contribution can be modeled as a power fluctuation:

11 $$ \sqrt{< {\Phi}_{\text{{\small temp}}}>^{2} }= \sqrt{4 k_{B} K {T^{\ast}}^{2}} , $$

where *K* is the thermal conductance of the device.

**Fig. 5:**
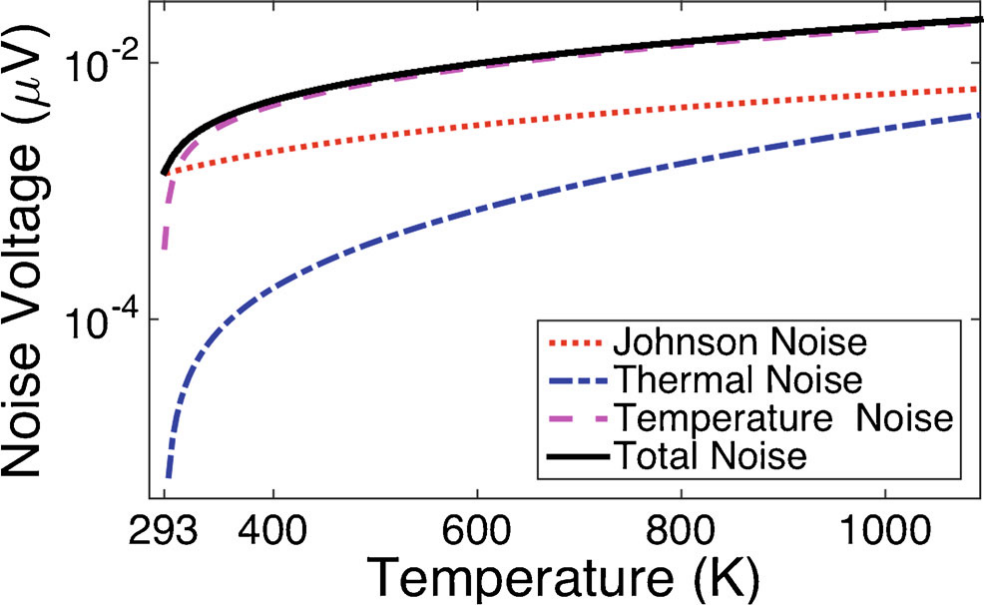
Semilog representation of the contribution to the noise vs. temperature of three noise mechanism applicable to the distributed bolometer optical antenna. The *solid line* represents the total noise of the device

The previous noise sources combine in quadrature and produce a variation of the signal voltage, *V*_*s*_. Johnson voltage can be directly included within this quadrature addition. For thermal noise and temperature noise, we use Eq. (8) to compute the noise voltage when the power given by Eqs. (10) and (11) are injected in the device. These contributions to noise are plotted in Fig. 5 where we can check that temperature noise as the most predominant source.

Once noise is evaluated, we estimate a signal-to-noise ratio (SNR) for the dipole antenna. The map of Fig. 4b shows SNR as a function of temperature and wavelength. Additionally, our results for the signal and noise of the device establish a condition for the stability of the bias voltage source. From Fig. 1b, we have evaluated that temperature changes at a maximum rate of 3K/mV at the highest bias voltage level (when temperature is around 1000 K). Conversely, at low temperature (close to 293 K), the variation of the bias voltage per degree reaches its maximum value of about 30 mV/K. If the bias voltage varies from 0 to around 1.5 V, we may assure a stability in this value in the order of 0.1 mV for a variation in temperature lower than 1K at the highest range in *V*_*b*_. This stability requirement can be relaxed if temperature is closer to room temperature. These requirements are easily fulfilled by a laboratory bias source. It is key that the measurement of the signal, *V*_*s*_, is done with synchronous lock-in techniques to sense the variation of the voltage, *V*_*s*_, with respect to the bias, *V*_*b*_, allowing the acquisition of very low signals. Figure 4a shows signal values in the order of tenths of microvolts that are easily detected with a lock-in measurement method.

## Spectrum Evaluation

In this section, we apply the previous results to obtain the spectral composition of an optical signal. Let us consider an arbitrary spectral irradiance $\mathcal {I} (\lambda )$. If the response of the antenna is described as $\mathcal {R}(\lambda , T)$, the signal obtained from the antenna is as follows:

12 $$ V_{s} (T)= {\int}_{\lambda_{\text{{min}}}}^{\lambda_{\text{{max}}}} {\mathcal R} (\lambda, T) {\mathcal I} (\lambda) d \lambda . $$

Therefore, temperature settings and its stability are important to obtain the desired spectrum composition. In a practical device, the bias voltage changes and establishes the operational temperature *T* ^∗^. Calibration must precede the use of the device. In a first approach, we consider that the voltage changes in discrete steps setting the temperature at preselected values, $ T^{\ast }_{i}$, where *i* runs from 1 to *N*, being *N* the number or steps in the biasing voltage. At the same time, we consider the spectral irradiance sampled at some specific wavelengths, *λ*_*j*_, where *j* runs from 1 to *M*, being *M* the number or wavelengths selected in the spectral sampling. In this situation, Eq. (12) becomes a matrix relation:

13 $$ \mathbf{V_{s} }(T) = \mathbf{R} (\lambda, T) \times \mathbf{I}(\lambda), $$

where *V*_*s*_ = (*V*_*s*_(*T*_1_),⋯ , *V*_*s*_(*T*_*i*_),⋯ , *V*_*s*_(*T*_*N*_))^*T*^ is a *N* dimensional column vector (superscript ^*T*^ means transposition), $\mathbf {I}= ({\mathcal I}(\lambda _{1}), \cdots , {\mathcal I}(\lambda _{j}), \cdots , {\mathcal I}(\lambda _{M}) )^{T}$ is a *M* dimensional column vector, and **R**(*λ*, *T*) is a *N* × *M* matrix containing the response of the device. After inverting Eq. (13), we obtain the input spectral irradiance, **I**(*λ*). This mathematical process implies important considerations about noise and the goodness of the inverted result.

### Retrieval of the Spectral Information

Figure 4 represents a smooth dependence of the signal and SNR for the case treated here. We have obtained it from multiphysics simulation of the device. However, in an actual device, some discrepancies may arise and produce a response deviating from the modeled case, needing calibration to validate the device. From an statistical point of view, spectral response affected by noise can be modeled as a multinormal distribution for each temperature.

As we mentioned, the results of the inversion of Eq. (13) are strongly dependent on the characteristics of the response matrix, **R**(*λ*, *T*), and the level of noise. The main issue for the retrieval of the input spectral content is related with the rank of the response matrix. For a square *N* × *N* matrix having a rank *N*, this is not a problem. However, this could not be the case due to the characteristics of the simulation or the results obtained after calibrating the device. When the matrix is not a square matrix, the spectrum is obtained using the generalized inverse:

14 $$ \mathbf{I}= \left( \mathbf{R}^{T} \mathbf{R} \right)^{-1} \mathbf{R}^{T} (\mathbf{V} - \mathbf{n} ),  $$

where **n** is the value of the noise. Matrix inversion can be ill-conditioned if the singular values of the matrix are close to zero. To solve this issue, we use a regularization procedure presented by Tikhonov [27]. This regularization inserts a parameter, *β*, that adds a constant to each singular value of the previous square matrix **R** ^*T*^ **R**. Then, Eq. (14) becomes

15 $$ \mathbf{I}= \left( \mathbf{R}^{T} \mathbf{R} + \beta^{2} \mathbf{1} \right)^{-1} \mathbf{R}^{T} (\mathbf{V} - \mathbf{n} ). $$

The question now is how to set the regularization parameter *β*. If *β* is very small, then the procedure does not provide a good solution because noise is enhanced. However, if this parameter is very large, all the eigenvalues of the matrix would be the same and results are not correct. The solution is to find a *β* value that provides a stable inversion of the matrix and retrieves a spectral irradiance close enough to the actual one. To do so, we propose a metric that compares the result obtained from Eq. (15), **I**_*β*_, with the actual spectrum impinging on the antenna, **I**_0_. This parameter is defined as the mean value of the square of the difference between the solution and the actual spectral, averaged over a collection of noise realizations. The result is

16 $$ i_{\beta}^{2}= \left\langle | \mathbf{I}_{\beta} - \mathbf{I}_{0} |^{2} \right\rangle = \mathbf{V}^{T} \mathbf{R}_{\beta}^{T} \mathbf{R}_{\beta} \mathbf{V} + \left\langle \mathbf{n}^{T} \mathbf{R}_{\beta}^{T} \mathbf{R}_{\beta} \mathbf{n} \right\rangle - \mathbf{I}_{0}^{T} \mathbf{I}_{0}, $$

where 〈〉 means sample averaging. This deviation, *i*_*β*_, defines a relative error: Γ_*β*_ = *i*_*β*_/|**I**_0_|, where |**I**_0_| is the Euclidean norm of vector *I*_0_. The goal now is to choose a value of *β* that minimizes this relative error, Γ_*β*_. Unfortunately, from this methodology, there is an intrinsic dependence with the type of spectrum that arrives to the detector, **I**_0_. To check the validity of the method, we have applied it to two types of spectra:

17 $$\begin{array}{@{}rcl@{}} {\mathcal I}(\lambda)_{\text{{\small gaussian}}} & = & a_{1} \exp{ \left[- \left( \frac{\lambda - \lambda_{c,1} }{ {\Delta} \lambda_{1}} \right)^{2} \right] }\\ &&+ a_{2} \exp{ \left[- \left( \frac{\lambda - \lambda_{c,2} }{\Delta \lambda_{2} } \right)^{2} \right] }, \end{array} $$

18 $$\begin{array}{@{}rcl@{}} {\mathcal I}(\lambda)_{\text{{\small BB}}} & = & \frac{2hc^{2}}{\lambda^{5}} \frac{1}{\exp{ \frac{hc}{\lambda k_{B}T} -1}} .\end{array} $$

**Fig. 6:**
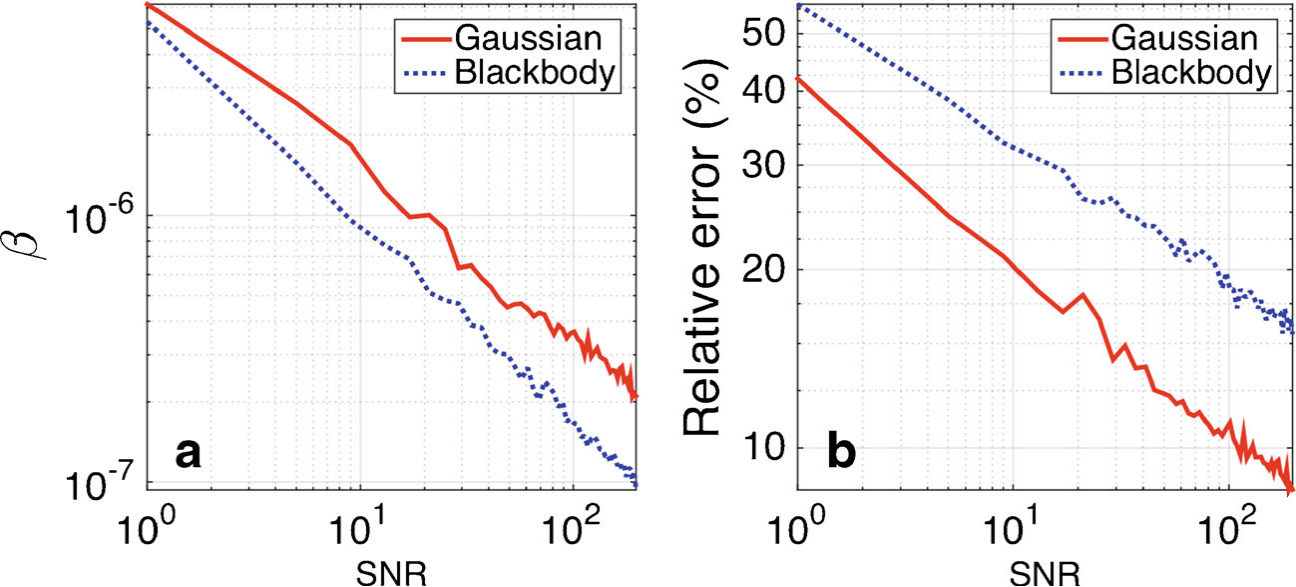
**a** Optimum value of the regularization parameter *α* for two types of spectra and different levels of noise. **b** Relative error, Γ_*β*_, of the retrieved spectrum with respect to the actual one as a function of SNR

The first spectrum, ${\mathcal I}(\lambda )_{\text {{\small gaussian}}}$, corresponds with two mixed Gaussians where *a*_1_ = 0.5, *a*_2_ = 0.8, *λ*_*c*,1_ = 10.6μm, *λ*_*c*,2_ = 8.5μm, Δ*λ*_1_ = 0.5μm, and Δ*λ*_2_ = 1.3μm. The second spectrum, ${\mathcal I}(\lambda )_{\text {{\small BB}}}$, is for a blackbody radiation at *T* = 300K. SNR was varied from 1 to 200, and 100 samples have been realized to properly average the results. Figure 6 represents the values of the optimum regularization parameter, *β*, and the minimum relative error, Γ_*β*_, as a function of the SNR ratio for the two types of spectra. In both cases, the dependence with SNR is quite similar and the values of *β* and Γ_*β*_ are in the same order of magnitude. SNR can be increased by extending the integration time in the measurement and taking more samples. For example, as far as SNR is proportional to $\sqrt {m}$, being *m* the number of samples, an increase in 1 order of magnitude of SNR can be attained taking 100 times more samples. As long as the characteristic period due to thermal inertia is in the range of the μs, for stable spectra it is still possible to time-average more measurements and obtain better accuracy.

Using this procedure and knowing the type of spectral composition under analysis, we tune the retrieval of the spectral information by selecting the regularization parameter, *β*, applicable to a given case. Also, the results can be qualified in terms of the relative error, Γ_*β*_, obtainable for each case. The main drawback of this method is the dependence with the type of spectra under analysis.

### Monochromatic Spectrum Measurement

**Fig. 7:**
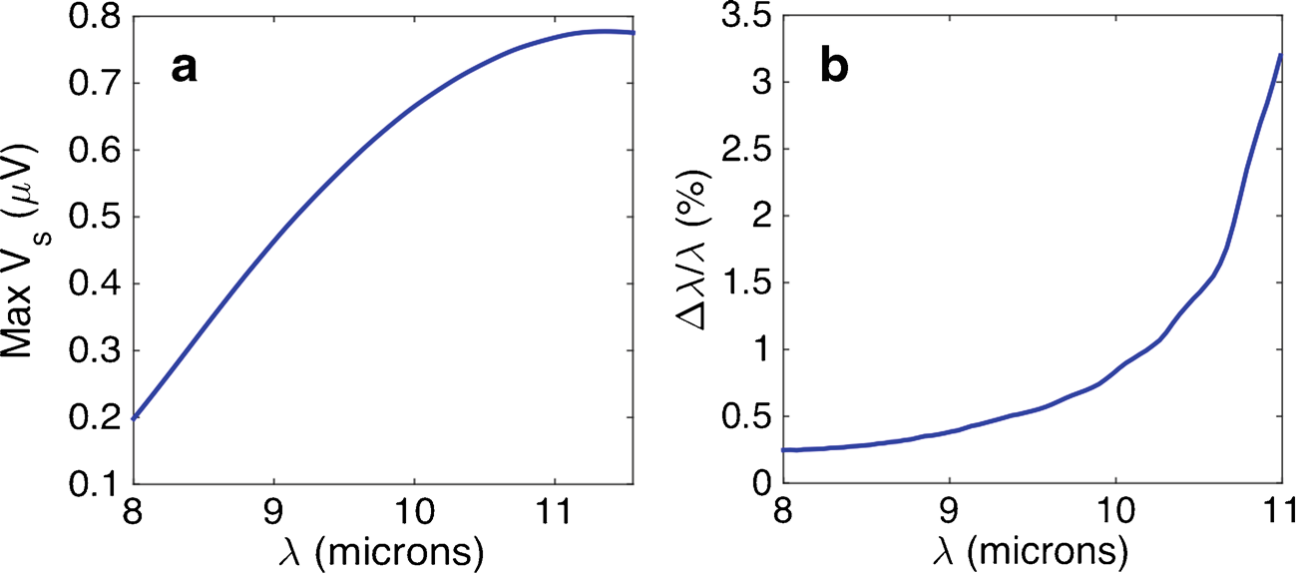
**a** Signal voltage at the maximum for each wavelength. This plot can be obtained from Fig. 4b at those points represented by the *black line* in the map. **b** Relative error in wavelength as a function of wavelength

We have seen that regularization procedures depend on the spectrum type. However, if we know that radiation is monochromatic, we can follow another method. Using Eq. (12), a monochromatic radiation at *λ*_0_ can be described as a delta function ${\mathcal I}(\lambda )=\delta (\lambda - \lambda _{0}$). In this case, the signal is proportional to the response ${\mathcal R}(\lambda _{0},T)$. This response has a maximum value at a temperature that is different for each input wavelength. The location of the maximum at each wavelength is plotted as a solid black curve in Fig. 4a. Maximum signal values can be given as a function of wavelength *V*_*s*,max_(*λ*), and are plotted in Fig. 7a. After calibration, determining the temperature of the maximum signal provides the value of *λ*_0_. However, noise is always present and has to be included to determine the accuracy of this maximum temperature value. Here, noise, *V*_*n*,max_, can be seen as the variation in signal, Δ*V*_*s*,max_. This condition can be written as

19 $$ V_{n,\text{{\small max}}}={\Delta} V_{s,\text{{\small max}}} = \left| \frac{\partial V_{s,\text{{\small max}}}} {\partial \lambda} \right| {\Delta} \lambda, $$

where Δ*λ* is the uncertainty in *λ* due to noise. Besides, *V*_*n*,max_ should be taken at the same points where *V*_*s*,max_(*λ*) is evaluated (along the black solid curve in Fig. 4a). Figure 7b shows the relative error, Δ*λ*/*λ*, that is expected from the measurements of monochromatic spectra with the proposed device. We may see that this relative error increases with *λ* because the maximum of the signal appears at higher temperatures when *λ* increases. Higher temperatures imply higher noise values and degrade spectral resolution. Relative error remains below 1 % when *λ*∈(8,10)μm. For monochromatic detection at a limited spectral range, the design of the element can be customized for better performance requiring less temperature increase.

## Conclusions

In this paper, we have evaluated the current and the dissipated power as a function of temperature and wavelength for a single dipole antenna. The variation of the response of the antenna when changing the temperature makes possible the spectral tuning of optical antennas. Biasing is able to heat the antenna and allows an electronic driving of its spectral response. The device is made of nickel and placed on a Si wafer coated with a SiO _2_ insulation layer. Our calculations are made within the thermal and electromagnetic domains by using a multiphysics finite-element method package. Current flowing through the feed point of the dipole determines the signal for a MIM antenna-coupled device. For a distributed bolometer optical antenna, the variable of interest is the power dissipated within the dipole’s volume. To simplify the analysis, we have focused on the bolometric transduction mechanism. Although spectra are not directly obtained from the signal given by the device, the results of this analysis can be used to create an optical antenna device able to determine the spectrum of infrared radiation impinging on the resonant structure. The spectral range considered in this study corresponds with the long wave infrared window (8–12 μm).

We have also analyzed the role of noise contributions applicable to the device. From the noise model, we have obtained SNR as a function of wavelength and temperature. Noise limits the quality and accuracy of the spectral information retrieved from the signal. The determination of the spectrum involves the inversion of the relation between spectral irradiance and signal. To do this in presence of noise, it is necessary to apply regularization techniques. This procedure is dependent on the type of spectrum incident on the device. However, the values of the regularization parameter and the relative error do not vary too much for the two cases analyzed, showing a good behavior of the method. Moreover, as long as the temporal response of the device is fast, it is possible to extend acquisition time, improve SNR, and obtain a lower relative error. For monochromatic spectra, the proposed device can determine its wavelength. In this case, taking into account noise, spectral resolution is also obtained and given as a relative error in the determination of wavelength. This error increases with wavelength and is as low as 1 % for *λ*∈(8,10)μm.

We conclude that it is possible to use a single optical antenna driven by temperature to determine the spectral content of a light beam or even to find the central wavelength of a monochromatic spectrum in the infrared. This determination maintains very high spatial resolution and polarization sensitivity inherently related with optical antennas.
